# The Tai Chi training for middle-aged and elderly patients with knee osteoarthritis: A protocol for systematic review and meta analysis

**DOI:** 10.1097/MD.0000000000020242

**Published:** 2020-05-15

**Authors:** Runyuan Ren, Guangjun Tang, Chenjian Tang, Jiayuan Zhang, Xiao Xiao, Qi Zhang

**Affiliations:** Chengdu university of traditional Chinese medicine, Chengdu, Sichuan province, China.

**Keywords:** KOA, meta-analysis, randomized controlled trial, systematic review, Tai Chi

## Abstract

**Background:**

Knee osteoarthritis (KOA) is a disabling joint disease with an increasingly prevalence among the older individuals. Tai Chi, one of the ancient meditative movements, has been recognized to have clinical benefits for KOA. We aim to evaluate the efficacy and safety of Tai Chi for patients with KOA through this systematic review.

**Methods:**

Five English databases (Cochrane Central Register of Controlled Trials (CENTRAL), MEDLINE, EMBASE, AMED, and CINAHL), 4 Chinese databases (CBM, CNKI, CQVIP, and Wanfang), and 5 clinical trial registration databases (ClinicalTrials.gov, ANZCTR, EU-CTR, ChiCTR, and ICTRP) will be searched from establishment of the database until November 31, 2019. Grey literature will be searched in SIGLE, Grey Net, Microsoft Academic, Google Scholar, Open Aire, World Wide Science.org, and WorldCat. There will be no restrictions on language. The randomized controlled trials of Tai Chi training for patients with KOA will be included. The primary outcome will be assessed according to the Western Ontario and McMaster Universities Osteoarthritis Index (WOMAC). Meta-analysis will be conducted with the use of RevMan 5.3. The specific process will refer to the Cochrane Handbook 5.1 for Systematic Review.

**Results:**

High-quality synthesis of current evidence on the efficacy and safety of Tai Chi training for KOA will be provided in this study.

**Conclusion:**

This systematic review aims to present evidence for whether Tai Chi training is an effective intervention which can improve both physical condition and life quality in patients suffering KOA.

## 1 Introduction

Osteoarthritis (OA), one of the most prevalent joint diseases among the elderly individuals,^[1]^ is a leading cause of disability in the United States^[2]^ and other developed countries.^[3]^ Since the mid-20th century, the disease has doubled in prevalence around the world.^[4]^ In 2010, the global age-standardized prevalence of knee OA was 3.8%.^[5]^ At least 19% adults aged 45 year and older suffering from KOA in the United States,^[5]^ while in China, the overall prevalence of symptomatic KOA was as high as 8.1% in 2012.^[6]^

Prior evidence indicated that KOA was proximately caused by the breakdown of joint tissues from mechanical loading^[7]^ and inflammation.^[8]^ However, the deeper underlying causes of KOAs high prevalence remain unclear. Some previous studies showed that KOA was primarily a disease of aging and overweight,^[9]^ so the aging of the population and the obesity epidemic might explain the increasing prevalence of this disease.^[10]^ Besides, the common risk factors of KOA also include some well-studied mismatch diseases that has shown an increasing prevalence in recent decades, such as hypertension, atherosclerotic heart disease, and type 2 diabetes.^[11,12]^

Physical impairment, pain, and psychological distress are often intertwined in individuals with KOA. Previous researches indicated that because of the chronic pain and poor physical function of knees, patients with KOA were inclined to have worse sleep quality,^[13,14]^ higher depressed mood,^[15]^ and be more anxious^[16]^ than healthy people. Besides joint discomfort, long-term sleep disturbances and negative emotions might directly produce deleterious effects on individuals physical and mental health, as well as reduce the quality of life.^[17]^

Nonpharmacologic treatments for KOA, mainly involving patient education, physical therapy, therapeutic exercise, and weight control^[18,19]^ are recommended due to the cost issues and patient concerns regarding analgesic-related adverse effects causing by analgesic medications.^[20,21]^

Tai Chi is a common type of palliative low-impact and aerobic exercise which combines gentle movement, meditation, as well as rhythmic breathing.^[22]^ As a traditional Chinese mind-body exercise, Tai Chi could bring beneficial effects for KOA individuals by improving the balance ability, muscle strength, cardiopulmonary function and sleep quality,^[23,24,25]^ and relieving psychological problems such as depression, anxiety, and tension.^[26]^

Since the clinical reports on Tai Chi training for KOA gradually increased in the last few years, some systematic reviews have been conducted. To the best of our knowledge, previous systematic reviews focused on the evidence to support the effects of Tai Chi on pain relief and physical function improvement in the patients with KOA.^[27,28]^ Only 1 research^[29]^ attached importance to mental effects, but the articles were searched just in English databases which was not comprehensive. High-quality evidence to indicate the mental and physical efficacy and safety of Tai Chi training for KOA is still lacking. Therefore, we conduct this systematic review to objectively evaluate whether Tai Chi training is a more effective and safer therapy for individuals suffering KOA.

## 2 Methods

### 2.1 Registration

This systematic review protocol has been registered on PROSPERO as CRD 42020106645. In this paper, the protocol will be performed using the methods introduced in the Cochrane Handbook 5.1 for Systematic Reviews of Intervention and reported according to the PRISMA-P guidelines. If we will refine procedures described in this protocol, we will document the amendments in the PROSPERO database and disclose them in future publications related to this meta-analysis.

### 2.2 Eligibility criteria for considering studies

#### 2.2.1 Types of studies

The RCTs are eligible. There are no restrictions on languages. Articles repeatedly published should be excluded.

#### 2.2.2 Types of participants

Inclusion criteria for participants include:

1. having a clinical diagnosis of KOA according to any recognized criteria, diagnosed through the use of knee radiographs and physical examination, and
2. being 45 years of age or older of any gender or ethnic background.

Exclusion criteria include:

1. having other orthopedic problems of the hip, knee, or ankle, or
2. having a neurological disease (e.g., Parkinsons, dementia, vertigo, or cerebral apoplexy).

Baseline is uniform for all participants in each RCT.

#### 2.2.3 Types of interventions

Participants in experimental group should be treated with Tai Chi training which has no restriction on types or training periods. Tai Chi training combined with other forms of therapy or other rehabilitation programs should be excluded.

The control group should adopt one of the following treatment methods: Health education, self-help program, other exercise, physical therapy, placebo, or no treatment.

#### 2.2.4 Types of outcome measures

##### 2.2.4.1 Primary outcome

Total score of the Western Ontario and McMaster Universities Osteoarthritis Index (WOMAC). The higher the global WOMAC score is, the worse the function of knees is.

##### 2.2.4.2 Secondary outcome

1. Frequency and nature of adverse events,
2. Physical performance tests include: changes in gait kinematic measures, Short Physical Performance Battery (SPPB), Lower Extremity Functional Scale (LEFS), knee joint proprioception, Visual Analogue Scale, knee range of motion (ROM), Berg Balance Scale (BBS), Lequesne&Mery Index, and Timed “up and go” test, and
3. Measurements to assess quality of life include: the Pittsburgh Sleep Quality of Index (PSQI), short form (36) health survey (SF-36), and the Five Facet Mindfulness Questionnaire (FFMQ).

### 2.3 Search methods for identifying the studies

#### 2.3.1 Data sources and searches

Five English databases (Cochrane Library, MEDLINE, EMBASE, CINAHL, and AMED), 4 Chinese databases (CBM, CNKI, CQVIP, and Wanfang), and 5 clinical trial registration databases (ClinicalTrials.gov, ICTRP, ChiCTR, EU-CTR, and ANZCTR) will be searched from establishment of the database until November 31, 2019. Grey literature will be searched in SIGLE (System for Information on Grey Literature in Europe), Grey Net, Microsoft Academic, Google Scholar, Open Aire, World Wide Science.org, and WorldCat. There are no restrictions on languages. The key search terms are [(“Tai ji” OR “Tai-ji”OR “Tai Chi” OR “Chi, Tai” OR “Tai Ji Quan” OR “Ji Quan, Tai” OR “Quan, Tai Ji” OR “Taiji” OR “Taijiquan” OR “T’ai Chi” OR “Tai Chi Chuan”) AND (“Osteoarthritis, Knee” OR “Knee Osteoarthritides” OR “Knee Osteoarthritis” OR “Osteoarthritides, Knee” OR “Osteoarthritis Of Knee∗” OR “Knee∗, Osteoarthritis Of”)]

### 2.4 Study selection and data extraction

**Figure 1:**
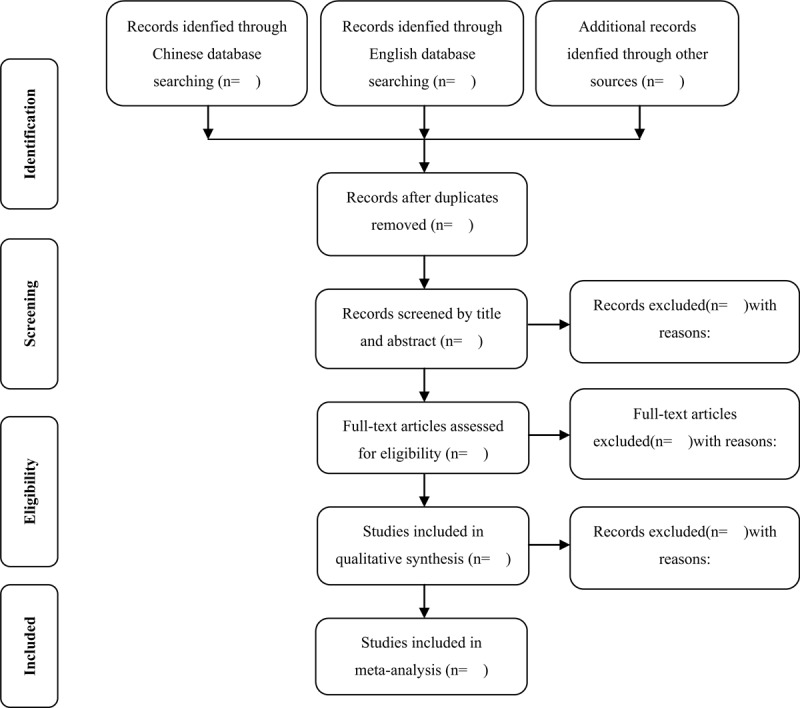
Flow diagram of study selection process.

Two researchers (Runyuan Ren and Guangjun Tang) search and screen the studies independently by finding duplications, excluding irrelevant titles and abstracts, and then selecting eligible studies by reviewing full texts. The inclusion and exclusion criteria are listed above. Reasons for exclusion should be noted. The third reviewer (Chenjian Tang) verified all information. Any disagreements should be solved by discussion until a consensus was reached. The specific process of study selection is shown in Figure 1.

Data extraction will be performed by 2 review authors (Runyuan Ren and Chenjian Tang) independently. The data extraction is conducted by a standard form, which contained First author, characteristics of the study type (date of publication, country, arm), participants (sample size, mean age, gender, duration of insomnia history), diagnostic instrument, intervention details, outcomes, follow-up, adverse events, and reported results. Finally, the data obtained by the 2 reviewers will be checked each other. If the data is incomplete, the original author will be contacted.

### 2.5 Assessment of risk of bias

Two researchers (Runyuan Ren and Chenjian Tang) assess the risk of bias independently, using a collaboration tool recommended by the Cochrane Handbook 5.1.^[30]^ Seven domains should be evaluated, including random sequence generation, allocation concealment, blinding of participants and personnel, blinding of outcome assessment, incomplete outcome data, selective reporting, and other bias. Disagreement will be settled by discussion.

### 2.6 Data analysis

#### 2.6.1 Date synthesis

Data analysis will be performed with Review Manager 5.3 software provided by the Cochrane Collaboration (www.cochrane.org). Continuous outcomes were presented as mean difference (MD)with 95% confidence interval (CI) between 2 groups, whereas dichotomous data were presented as relative risk (RR) with 95% CI. It is considered statistically significant when *P* < .01.

#### 2.6.2 Assessment of heterogeneity

The Chi-Squared test and *I*^2^ statistic are used to assess heterogeneity. The fixed-effect model is suitable to estimate the typical effect for studies with low heterogeneity (*I*^2^ < 50% or *P* > .10), whereas the random-effects model is used to assess the average distribution for studies with substantial unexplained heterogeneity (*I*^2^ ≥ 50% or *P* ≤ .10).

#### 2.6.3 Subgroup analysis and sensitivity analysis

Subgroup analysis and sensitivity analysis will also be employed to explore the possible causes of heterogeneity. Subgroup analysis will be based on possible factors that may lead to heterogeneity, such as intervention (different types of Tai Chi training), control (health education, self-help program, other exercise, physical therapy, placebo or no treatment), ages (middle-age, old), treatment duration, the quality of study, etc. Narrative synthesis will be considered if quantitative synthesis is not appropriate.

### 2.7 Assessment of publication bias

If more than 10 articles are included, publication bias will be analyzed by visual inspection of funnel plots. A symmetrical distribution of funnel plot data indicates that there is no publication bias.

### 2.8 Confidence in cumulative evidence

GRADE system will be used for assessing the strength of the body of evidence.^[31]^ According to the grading system, the quality of evidence will be rated high, moderate, low, and very low.

## 3 Discussion

KOA is a disabling joint disease with a high-prevalence among the middle-aged and old individuals. The pain, stiffness, and dysfunction of knees bring about poor sleep quality and negative moods involving depression, anxiety and tension, which directly reduce the quality of life. For cost issues and patient concerns regarding analgesic-related adverse effects causing by analgesic medications, Tai Chi training has been accepted by increasing number of patients suffered from KOA. As a traditional Chinese mind-body exercise, Tai Chi training has popularized especially in Asian countries. Since several recent clinical researches have focused on this promising treatment for KOA, it is necessary to perform a high-quality systematic review and meta-analysis. Therefore, this review is expected to provide rigorous and objective evidences of the efficacy and safety of Tai Chi training for KOA.

## Author contributions

**Conceptualization:** Runyuan Ren, Xiao Xiao, Qi Zhang.

**Data curation:** Runyuan Ren, Guangjun Tang, Chenjian Tang.

**Formal analysis:** Chenjian Tang, Runyuan Ren.

**Investigation:** Runyuan Ren.

**Methodology:** Runyuan Ren, Chenjian Tang.

**Project administration:** Jiayuan Zhang, Qi Zhang.

**Resources:** Guangjun Tang.

**Software:** Runyuan Ren,Gu Innovative Research Group Project of the National Natural Science Foundation of China (CN) angjun Tang, Chenjian Tang, Jiayuan Zhang.

**Supervision:** Qi Zhang, Jiayuan Zhang.

**Validation:** Qi Zhang.

**Writing – original draft:** Runyuan Ren, Xiao Xiao, Qi Zhang.

**Writing – review and editing:** Runyuan Ren, Qi Zhang.

## References

[1] Brosseau L Taki J Desjardins B The Ottawa panel clinical practice guidelines for the management of knee osteoarthritis. Part one: introduction, and mind-body exercise programs. Clin Rehabil 2017;31:582–95. 28183188 10.1177/0269215517691083

[2] Murray CJ Atkinson C Bhalla K The state of US health, 1990-2010: Burden of diseases, injuries, and risk factors. JAMA 2013;310:591–606. 23842577 10.1001/jama.2013.13805 PMC5436627

[3] Vos T Flaxman AD Naghavi M Years lived with disability (YLDs) for 1160 sequelae of 289 diseases and injuries 1990-2010: A systematic analysis for the Global Burden of Disease Study 2010. Lancet 2012;380:2163–96. 23245607 10.1016/S0140-6736(12)61729-2 PMC6350784

[4] Wallace IJ Worthington S Felson DT Knee osteoarthritis has doubled in prevalence since the mid-20th century. Proc Natl Acad Sci USA 2017;114:9332–6. 28808025 10.1073/pnas.1703856114 PMC5584421

[5] Lawrence RC Felson DT Helmick CG Estimates of the prevalence of arthritis and other rheumatic conditions in the United States. Part II. Arthritis Rheum 2008;58:26–35. 18163497 10.1002/art.23176 PMC3266664

[6] Cross M Smith E Hoy D The global burden of hip and knee osteoarthritis: estimates from the global burden of disease 2010 study. Ann Rheum Dis 2014;73:1323–30. 24553908 10.1136/annrheumdis-2013-204763

[7] Felson DT Osteoarthritis as a disease of mechanics. Osteoarthritis Cartilage 2013;21:10–5. 23041436 10.1016/j.joca.2012.09.012 PMC3538894

[8] Robinson WH Lepus CM Wang Q Low-grade inflammation as a key mediator of the pathogenesis of osteoarthritis. Nat Rev Rheumatol 2016;12:580–92. 27539668 10.1038/nrrheum.2016.136 PMC5500215

[9] Abbasi J Can exercise prevent knee osteoarthritis? JAMA 2017;318:2169–71. 29167894 10.1001/jama.2017.16144

[10] Heidari B Knee osteoarthritis prevalence, risk factors, pathogenesis and features: part I. Caspian J Intern Med 2011;2:205–12. 24024017 PMC3766936

[11] Lieberman DE The Story of the Human Body: Evolution, Health, and Disease. New York:Pantheon; 2013. 27875612

[12] Zhuo Q Yang W Chen J Metabolic syndrome meets osteoarthritis. Nat Rev Rheumatol 2012;8:729–37. 22907293 10.1038/nrrheum.2012.135

[13] Chen CJ McHugh G Campbell M Subjective and objective sleep quality in individuals with osteoarthritis in Taiwan. Musculoskelet Care 2015;13:148–59. 10.1002/msc.1094 25491038

[14] Hawker GA French MR Waugh EJ The multidimensionality of sleep quality and its relationship to fatigue in older adults with painful osteoarthritis. Osteoarthritis Cartilage 2010;18:1365–71. 20708004 10.1016/j.joca.2010.08.002

[15] Wioletta Tuszyńska-Bogucka Tomasz Psychosocial generalised resistance resources and clin-ical indicators of patients suffering from osteoarthritis at the institute of rural health in Lublin, Poland. Ann Agri Environ Med 2015;22:380–4. 10.5604/12321966.1152098 26094542

[16] Lee AC Driban JB Price LL Responsiveness and minimally important differences for 4 patient-reported outcomes measurement information system short forms: physical function, pain interference, depression, and anxiety in knee osteoarthritis. J Pain 2017;18:1096–110. 28501708 10.1016/j.jpain.2017.05.001 PMC5581239

[17] Pickering ME Chapurlat R Kocher L Sleep disturbances and osteoar-thritis. Pain Pract 2016;16:237–44. 25639339 10.1111/papr.12271

[18] Adams T Band-Entrup D Kuhn S Physical therapy management of knee osteoarthritis in the middle-aged athlete. Sports Med Arthrosc Rev 2013;21:2–10. 23314262 10.1097/JSA.0b013e318272f530

[19] Felson DT Osteoarthritis of the knee. N Engl J Med 2006;354:841–8. 16495396 10.1056/NEJMcp051726

[20] Sale JEM Gignac M Hawker G How “bad” does the pain have to be? A qualitative study examining adherence to pain medication in older adults with osteoarthritis. Arthritis Rheum 2006;55:272–8. 16583418 10.1002/art.21853

[21] Solomon Daniel H The comparative safety of analgesics in older adults with arthritis. Arch Intern Med 2010;170:1968–76. 21149752 10.1001/archinternmed.2010.391

[22] Shengelia R Parker SJ Ballin M Complementary therapies for osteoarthritis: are they effective? Pain Manag Nurs 2013;14:e274–88. 24315281 10.1016/j.pmn.2012.01.001 PMC3857560

[23] Vallabhajosula S Roberts BL Hass CJ Tai Chi intervention improves dynamic postural control during gait initiation in older adults: a pilot study. J Appl Biomech 2014;30:697–706. 25010527 10.1123/jab.2013-0256

[24] Lee MS Pittler MH Ernst E Tai chi for osteoarthritis: a systematic review. Clin Rheumatol 2008;27:211–8. 17874172 10.1007/s10067-007-0700-4

[25] Lü J Huang L Wu X Effect of Tai Ji Quan training on self-reported sleep quality in elderly Chinese women with knee osteoarthritis: a randomized controlled trail. Sleep Med 2017;33:70–5. 28449910 10.1016/j.sleep.2016.12.024

[26] Zhang L Layne C Lowder T A review focused on the psychological effectiveness of Tai Chi on different populations. Evid Based Complement Alternat Med 2012;2012:1–9. 10.1155/2012/678107 PMC3140024 21792371

[27] Lauche R Langhorst J Dobos G A systematic review and meta-analysis of Tai Chi for osteoarthritis of the knee. Complement Ther Med 2013;21:396–406. 23876571 10.1016/j.ctim.2013.06.001

[28] Ye J Cai S Zhong W Effects of Tai Chi for patients with knee osteoarthritis: a systematic review. J Phys Ther Sci 2014;26:1133–7. 25140112 10.1589/jpts.26.1133 PMC4135213

[29] Chang WD Chen S Lee CL The effects of tai chi chuan on improving mind-body health for knee osteoarthritis patients: a systematic review and meta-analysis. Evid Based Complement Alternat Med 2016;2016:1–0. 10.1155/2016/1813979 PMC5011213 27635148

[30] Higgins JP Green S Cochrane handbook for systematic review of interventions version 5.1. 0 [updated March 2011]. Cochrane Collab 2011.

[31] Guyatt GH Oxman AD Vist GE GRADE: an emerging consensus on rating quality of evidence and strength of recommendations. BMJ 2008;336:924–6. 18436948 10.1136/bmj.39489.470347.AD PMC2335261

